# The value of metagenomic next-generation sequencing with different nucleic acid extracting methods of cell-free DNA or whole-cell DNA in the diagnosis of non-neutropenic pulmonary aspergillosis

**DOI:** 10.3389/fcimb.2024.1398190

**Published:** 2024-07-29

**Authors:** Xiaomin Cai, Chao Sun, Huanhuan Zhong, Yuchen Cai, Min Cao, Li Wang, Wenkui Sun, Yujian Tao, Guoer Ma, Baoju Huang, Shengmei Yan, Jinjin Zhong, Jiamei Wang, Yajie Lu, Yuanlin Guan, Mengyue Song, Yujie Wang, Yuanyuan Li, Xin Su

**Affiliations:** ^1^ Department of Respiratory and Critical Care Medicine, Nanjing Drum Tower Hospital, Affiliated Hospital of Medical School, Nanjing University, Nanjing, China; ^2^ Department of Respiratory and Critical Medicine, Jinling Hospital, Affiliated Hospital of Medical School, Nanjing University, Nanjing, China; ^3^ Department of Respiratory and Critical Care Medicine, The Second Affiliated Hospital of Suzhou University, Suzhou, China; ^4^ Department of Respiratory and Critical Care Medicine, The Second Affiliated Hospital of Nanjing University of Chinese Medicine, Nanjing, China; ^5^ Department of Respiratory and Critical Care Medicine, Jiangsu Province Hospital, The First Affiliated of Nanjing Medical University, Nanjing, China; ^6^ Department of Respiratory and Critical Care Medicine, Affiliated Hospital of Yangzhou University, Yangzhou, China; ^7^ Department of Respiratory and Critical Care Medicine, Affiliated Hospital of Jiangsu University, Zhenjiang, China; ^8^ Department of Respiratory and Critical Care Medicine, Jinling Hospital, Nanjing Medical University, Nanjing, China; ^9^ Department of Research and Development, Hugobiotech Co., Ltd., Beijing, China

**Keywords:** metagenomic next-generation sequencing (mNGS), cell-free DNA, whole-cell DNA, non-neutropenic pulmonary aspergillosis, pulmonary aspergillosis

## Abstract

**Purpose:**

Metagenomic next-generation sequencing(mNGS) is a novel molecular diagnostic technique. For nucleic acid extraction methods, both whole-cell DNA (wcDNA) and cell-free DNA (cfDNA) are widely applied with the sample of bronchoalveolar lavage fluid (BALF). We aim to evaluate the clinical value of mNGS with cfDNA and mNGS with wcDNA for the detection of BALF pathogens in non-neutropenic pulmonary aspergillosis.

**Methods:**

mNGS with BALF-cfDNA, BALF-wcDNA and conventional microbiological tests (CMTs) were performed in suspected non-neutropenic pulmonary aspergillosis. The diagnostic value of different assays for pulmonary aspergillosis was compared.

**Results:**

BALF-mNGS (cfDNA, wcDNA) outperformed CMTs in terms of microorganisms detection. Receiver operating characteristic (ROC) analysis indicated BALF-mNGS (cfDNA, wcDNA) was superior to culture and BALF-GM. Combination diagnosis of either positive for BALF-mNGS (cfDNA, wcDNA) or CMTs is more sensitive than CMTs alone in the diagnosis of pulmonary aspergillosis (BALF-cfDNA+CMTs/BALF-wcDNA+CMTs vs. CMTs: ROC analysis: 0.813 vs.0.66, P=0.0142/0.796 vs.0.66, P=0.0244; Sensitivity: 89.47% vs. 47.37%, P=0.008/84.21% vs. 47.37%, P=0.016). BALF-cfDNA showed a significantly greater reads per million (RPM) than BALF-wcDNA. The area under the ROC curve (AUC) for RPM of *Aspergillus* detected by BALF-cfDNA, used to predict “True positive” pulmonary aspergillosis patients, was 0.779, with a cut-off value greater than 4.5.

**Conclusion:**

We propose that the incorporation of BALF-mNGS (cfDNA, wcDNA) with CMTs improves diagnostic precision in the identification of non-neutropenic pulmonary aspergillosis when compared to CMTs alone. BALF-cfDNA outperforms BALF-wcDNA in clinical value.

## 1 Introduction

Pulmonary Aspergillosis (PA) is a serious infectious fungal disease, commonly seen in immunocompromised patients (El-Baba et al., 2020). *Aspergillus fumigatus* is the predominant culprit, responsible for more than 70% of cases. Depending on the interaction between *Aspergillus* and individuals with varying immune status and underlying diseases, PA is classified as invasive pulmonary aspergillosis (IPA), allergic bronchopulmonary aspergillosis (ABPA) and chronic pulmonary aspergillosis (CPA). With the increasing use of corticosteroids or antimicrobials, an aging population, PA is not limited to immunosuppressed populations. High-risk hosts include individuals like those with chronic obstructive pulmonary disease (COPD) (Guinea et al., 2010) and critically ill patients (Taccone et al., 2015). In recent years, the morbidity of PA has been increasing. However, PA in non-neutropenic patients is hard to be recognized in the early stage due to the atypical clinical and radiological manifestations and limited sensitivity of traditional diagnostic methods. Thus, the mortality stays high. Current diagnostic tools, such as Aspergillus-specific IgG (Lu et al., 2023) and Pentraxin 3 (He et al., 2018) may have some value in the diagnosis of non-neutropenic PA, but we still need quicker and earlier detection methods for PA. Therefore, finding a sensitive, efficient, specific and less invasive method for early PA diagnosis is of great value of improving outcomes.

Since in 2014, when Charles Y. Chiu’s team (Wilson et al., 2014) used metagenomic Next-Generation Sequencing (mNGS) to detect *Leptospira* for the first time to confirm the diagnosis of meningitis, mNGS has emerged as a novel molecular diagnostic technique. Over the past few years, mNGS has been recognized as a comprehensive, rapid and accurate method in detecting infectious pathogens in the nervous, respiratory and blood system infection offering advantages such as rapid detection, non-bias and broad spectrum. Commonly used samples include sputum, bronchoalveolar lavage fluid (BALF) and blood. However, few reports have been published about the application of mNGS in the study of PA in non-neutropenic patients. Basing on the methods of extracting nucleic acid, whole-cell DNA (wcDNA) and cell-free DNA (cfDNA) are both applied widely. mNGS of cfDNA may cause host DNA degradation, potentially leading to the loss of cfDNA in the supernatant. Conversely, fragmenting cells without degrading host DNA during the extraction of wcDNA from BALF samples can increase human DNA release. Determining the appropriate sample processing technique for clinical settings, but there are few reports on the clinical value of these two sample processing technologies.

Therefore, we performed this clinical study to analyze the pathogenicity of non-neutropenic patients with suspected PA in terms of sample type and nucleic acid extraction method. Our aims are to analyze the pathogenic profile in detail and to systematically evaluate the efficacy of mNGS in the diagnosis of PA.

## 2 Materials and methods

### 2.1 Study design and patient population

From March 2022 to October 2022, patients with suspected PA in non-neutropenic admitted to the Department of Respiratory and Critical Care Medicine of Nanjing Jinling Hospital, Nanjing Drum Tower Hospital, Nanjing First Hospital, Jiangsu Province Hospital, affiliated Hospital of Yangzhou university, Affiliated Hospital of Jiangsu university, Jiangsu Second Chinese Medicine Hospital.

Patients were eligible for enrollment if they were (1) age≥ 18 years; (2) including but not limited to: patients with underlying diseases, such as COPD, diabetes, and the use of corticosteroids; (3) have respiratory symptoms, like fever, cough, that have failed to respond to treatment with broad-spectrum antibacterial medication; (4) computed tomography (CT) showing lesions such as pulmonary nodules, infiltrative shadows or cavities. Patients were excluded from the study based on the following criteria: (1) age <18 years; (2) neutropenia (absolute neutrophil count <0.5×10^9^/L).

The patients were finally classified as the PA (including IPA and CPA) and non-PA groups. The clinical diagnosis of PA and whether the microorganism detected was pathogenic or colonizing was made by two senior pulmonologists based on host risk factors, clinical symptoms, chest computed tomography, laboratory findings, and response to treatment. The diagnostic criteria for IPA and CPA were mainly referred to the guidelines of the 2020 EORTC/MSGERC,2016 IDSA, 2017 ESCMID/ERS/ECMM (Denning et al., 2016; Patterson et al., 2016; Ullmann et al., 2018; Donnelly et al., 2020). Proven IPA requires a positive *Aspergillus* in sterile body fluids or tissues. The probable IPA needs the combination of (1) host factors like COPD, diabetes; and (2) clinical symptoms like fever, cough; and (3) CT showing lesions with or without a halo sign, infiltrative shadows, or cavities; and (4) microbiological evidence included positive results for *Aspergillus* culture or PCR, single Galactomannan (GM) test ≥1.0, or single serum/plasma GM ≥0.7 with BALF GM ≥0.8. The possible IPA needs at least one of the host factors and clinical features. CPA diagnosis relies on (1) clinical symptoms, like cough, sputum, or fever; (2) CT imaging like cavitation, fugal ball; and (3) *Aspergillus* culture positive or immunological response to *Aspergillus* (positive Aspergillus IgG or precipitin test). The disease has been present for at least 3 months.

BALF samples taken from patients with suspected PA underwent mNGS of cfDNA, mNGS of wcDNA, and conventional microbiological tests (CMTs). The CMTs included the GM test, culture, and smear microscopic for the bacteria and fungi, and smear microscopic for tuberculosis. Physical information and clinical details were investigated. The remaining BALF sample from each of the enrolled patients was collected into a 3 mL sterile tube and delivered to Hugobiotech (Hugobiotech, Beijing, China) immediately for mNGS of cfDNA and wcDNA(The minimum total volume of BALF required for the each experiment was 1.5 mL). The remaining 5 mL Blood samples from 8 enrolled patients were collected into a vacuum blood collective tube and delivered to Hugobiotech (Hugobiotech, Beijing, China) at room temperature and immediately for mNGS of cfDNA.

Concurrently, the remaining 3ml BALF from 62 of enrolled patients was collected into sterile tubes and immediately sent to KingMed (Guangzhou, China) for target next generation sequencing (tNGS) detection.

### 2.2 GM test and culture

The hospital laboratories performed the GM test using the double-sandwich enzyme-linked immunosorbent assay (Bio-Rad Laboratories). Samples of appropriately collected bronchoalveolar lavage fluid (BALF), comprising more than 10 mL, were cultured using CHROMagar and incubated at 35°C for three days.

### 2.3 mNGS detection

#### 2.3.1 Nucleic acid extraction

Based on its manual, cfDNA and wcDNA were extracted from clinical samples using QIAamp DNA Micro Kit (QIAGEN, Hilden, Germany). For cfDNA extraction, the supernatant of the sample is taken after centrifugation. For wcDNA, the sample is extracted directly without centrifugation. Using Qubit 3.0 Fluoremeter (Invitrogen, Q33216) and agarose gel electrophoresis (Major Science, UVC1–1100) check DNA concentration and quality.

#### 2.3.2 Library generation and sequencing

DNA library construction was carried out in line with the guidelines specified in the Qiagen library construction kit (QIAseq Ultralow Input Library Kit). Quality control of the library was conducted using both the Qubit Agilent 2100 Bioanalyzer (Agilent Technologies, Palo Alto, USA) and the 3.0 Fluoremeter (Invitrogen, Q33216). Eligible DNA libraries, labeled with different barcodes, were combined and sequenced using the SE75bp sequencing strategy and Illumina Nextseq 550 sequencing platform (Illumina, San Diego, USA).

#### 2.3.3 Bioinformation pipeline

After gaining the sequencing data, we filtered out splice sequences, low quality, low complexity, and shorter sequences to obtain high-quality data. Then, we use SNAP software to remove human-derived sequences that match the human reference database (hg38). Next, we aligned the remaining data to the microbial genome database using BWA-MEM (processing time is approximately 20 minutes, and the memory requirement is around 20G). Finally, we compared the remaining data with the microbial genome database using Burrow Wheeler Alignment. This database contains an extensive collection of microbial genomes from NCBI having more than 30,000 microorganisms, including 17,748 species of bacteria, 11,058 species of viruses, 1,134 species of fungi, and 308 species of parasites. Finally, the microbial composition in the sample was determined. The positive criteria for the mNGS result were set as follows (Gu et al., 2021):

- (1) To detect bacteria, fungi, and parasites, the sequencing coverage should be in the top 10 of all pathogens detected and not detected in the negative control (NTC), or the sample/NTC should have an RPM (reads per million mapped reads) ratio greater than 10.
- (2) For viruses, tuberculosis, and cryptococci, at least one specific sequence should be detected and not detected in the NTC, or the sample/NTC RPM ratio should be greater than 5.

### 2.4 tNGS detection

#### 2.4.1 Nucleic acid extraction and library preparation

The magnetic bead method is employed for the extraction of nucleic acids from samples.

The samples were subjected to amplification using ultra-multiplex PCR primers (a total of 153 respiratory pathogens (**Supplementary Table 1**) were analyzed with the aim of identifying highly conserved regions). This was achieved through the design of specific primers. The amplified PCR products were purified by magnetic beads and mixed with specific sequencing junction tags and a library amplifying enzyme for the second round of amplification. The products of the second round of amplification were purified by magnetic beads for the second time to obtain the libraries.

#### 2.4.2 Sequencing and bioinformatic analysis

Sequencing was conducted using the gene sequencer KM MiniSeqDx-CN. Following a comparison and analysis of the data from the sequencing machine with the database, the pathogenic situation in the samples was judged.

### 2.5 Statistical analysis

We used SPSS software (version 26, IBM Corp, Armonk, NY, USA), MedCalc (version 20.1), and Prism (version 9.5.1) for statistical analysis and drawing. Continuous variables were presented as mean ± SD. The t-test and Wilcoxon test for two group samples were used to compare the normal or abnormal distribution. We employed the Pearson chi-squared test and McNemar test (for paired data) or the Fisher’s exact test for categorical data. The specificity and sensitivity of detection methods in diagnosing PA were calculated (percentage with 95% confidence interval [CI]) and compared (chi-squared). Spearman’s r values were utilized to analyze their correlation. Furthermore, a receiver operating characteristic (ROC) curve was employed to determine the best test for identifying specific pathogens with true-positive results. The study implemented the Yoden index to establish the cut-off values for RPM in the ROC curve. A two-tailed P-value of less than 0.05 was considered statistically significant.

## 3 Results

### 3.1 Patient characteristics

A total of 71 suspected PA patients including 48 male and 23 female were enrolled in this study, **Table 1** displays their clinical features. Most patients have underlying diseases (97.2%, 69/71), such as lung cancer (7), COPD (9), and diabetes (18). The clinicians finally diagnosed 19 cases of PA (12 cases of IPA, and 7 cases of CPA) and 52 cases of non-PA (26 cases of bacterial infection, 4 cases of non-infectious diseases, 8 cases of Mycobacterium tuberculosis (MTB), 6 cases of other fungal diseases, 3 cases of non-tuberculous mycobacteria (NTM), and 1 case of Chlamydia psittaci pneumonia,4 cases of bacterial co-infections with other fungal).

**Table 1:** Clinical characteristics of PA and non-PA in non-neutropenic patients on admission.

Characteristic, n (%)	N=71	PA (n=19)	non-PA (n=52)	P-value
Male/Female	48/23	14/5	34/18	0.51
Age, mean (SD),years	61.17 ± 13.236	62.63 ± 7.974	60.63 ± 14.729	0.47
Smoking history	30 (42.25)	9 (47.37)	21 (40.38)	0.60
Drinking history	12 (16.90)	2 (10.53)	10 (19.23)	0.61
Admitted to ICU	18 (25.35)	5 (26.32)	13 (25.00)	1.00
Use of hormones for more than 3weeks within 60days				0.79
Vein/Oral	13 (18.31)	4 (21.05)	9 (17.31)	
Inhale	1 (1.41)	0	1 (1.92)	
Use of immunosuppressive agents within 30 days	8 (11.27)	1 (5.26)	7 (13.46)	0.59
Underlying diseases				
Hypertension	25 (35.21)	7 (36.84)	18 (34.62)	0.86
Diabetes	18 (25.35)	7 (36.84)	11 (21.15)	0.30
Bronchiectasis	16 (22.54)	7 (36.84)	9 (17.31)	0.16
Cerebrovascular disease	17 (23.94)	4 (21.05)	13 (25.00)	0.97
Pulmonary emphysema	15 (21.13)	4 (21.05)	11 (21.15)	1.00
Pulmonary tuberculosis	12 (16.90)	5 (26.32)	7 (13.46)	0.36
Cardiovascular disease	12 (16.90)	2 (10.53)	10 (19.23)	0.49
Other solid organ tumor (except lung cancer)	11 (15.49)	5 (26.32)	6 (11.54)	0.25
Chronic obstructive pulmonary disease	9 (12.68)	7 (36.84)	2 (3.85)	1.00
Interstitial Lung Disease	8 (11.27)	0	8 (15.38)	0.16
Chronic kidney diseases	7 (9.86)	2 (10.53)	5 (9.61)	1.00
Lung cancer	7 (9.86)	2 (10.53)	5 (9.61)	1.00
Hepatopathy	4 (5.63)	1 (5.26)	3 (5.77)	0.81
Congestive heart failure	3 (4.22)	0	3 (5.77)	0.56
Hematologic tumor	2 (2.82)	1 (5.26)	1 (1.92)	0.47
Organ transplantation	1 (1.41)	0	1 (1.92)	1.00
Clinical symptoms				
Fever	35 (49.30)	8 (42.11)	27 (51.92)	0.46
Cough	63 (88.73)	17 (89.47)	46 (88.46)	1.00
Shiver	5 (7.04)	1 (5.26)	4 (7.69)	1.00
Eexpectoration	61 (85.92)	17 (89.47)	44 (84.62)	0.89
Hemoptysis	13 (18.31)	5 (29.41)	8 (15.38)	0.56
Chest distress	33 (46.48)	11 (57.89)	22 (42.31)	0.24
Asthma/dyspnea	35 (49.30)	10 (52.63)	25 (48.08)	0.73
Chest pain	11 (15.49)	4 (21.05)	7 (13.46)	0.68
Chest computed tomography images				
Infiltration or exudation	45 (63.38)	12 (63.16)	33 (63.46)	0.98
Small nodule	23 (32.39)	8 (42.11)	15 (28.85)	0.32
Wedge-shaped and segmental or lobar consolidation	21 (29.58)	4 (21.05)	17 (32.69)	0.32
Cavitation sign	15 (21.13)	9 (47.37)	6 (11.54)	0.004
Multiple clump-like infiltrates or consolidations along the bronchovascular bundle	15 (21.13)	5 (26.32)	10 (19.23)	0.78
Tubercle	14 (19.72)	4 (21.05)	10 (19.23)	1.00
Tree bud sign	5 (7.04)	3 (15.79)	2 (3.85)	0.07
Mass	4 (5.63)	1 (5.26)	3 (5.77)	1.00
Air crescent sign	3 (4.22)	2 (10.53)	1 (1.92)	0.05
Halo sign	1 (1.41)	0	1 (1.92)	1.00
Pleural thickening				0.33
Unilateral	8 (11.27)	1 (5.26)	7 (13.46)	
Bilateral	18 (25.35)	7 (36.84)	11 (21.15)	
Pleural effusion				0.25
Unilateral	15 (21.13)	2 (10.53)	13 (25.00)	
Bilateral	11 (15.49)	2 (10.53)	9 (17.31)	

PA, Pulmonary aspergillosis; non-PA, non- Pulmonary aspergillosis.

### 3.2 Species distribution and consistency of microorganisms detected by BALF-cfDNA, BALF-wcDNA, and CMTs for suspected non-neutropenic pulmonary aspergillosis

**Figure 1:**
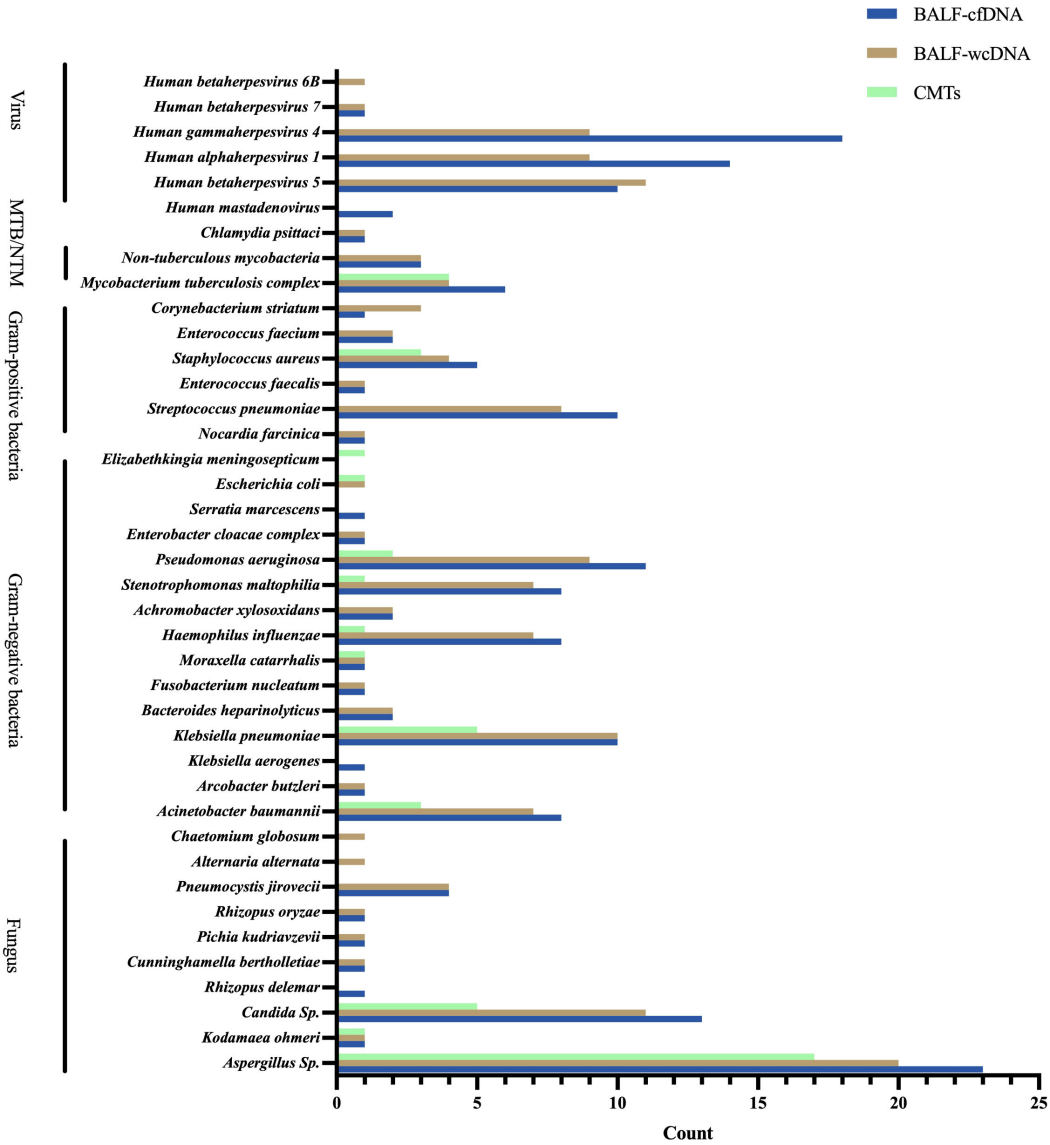
Species distribution of gram-positive bacteria, gram-negative bacteria, fungus, viruses, and chlamydia pasittaci detected by BALF-cfDNA, BALF-wcDNA, and CMTs. MTB, Mycobacterium tuberculosis; NTM, Non-tuberculous mycobacteria.

BALF-cfDNA detected 43 species (14 fungi, 19 bacteria, 6 viruses, 3 mycobacteria, 1 chlamydia), BALF-wcDNA detected 44 species (17 fungi, 18 bacteria, 5 viruses, 3 mycobacteria, 1 chlamydia), and CMTs detected 14 species (4 fungi, 9 bacteria,1 mycobacteria). As shown, BALF-mNGS (cfDNA, wcDNA) detected more species than CMTs. Five (*Rhizopus delemar, Klebsiella aerogenes, Serratia marcescens, Human mastadenovirus B, Human mastadenovirus C*) microorganisms were detected only by BALF-cfDNA. Six microorganisms (*Alternaria alternata, Candida parapsilosis Chaetomium globosum, Candida intermedia, Aspergillus glaucus, Human betaherpesvirus 6B*) were detected only by BALF-wcDNA. *Elizabethkingia meningosepticum* was identified by CMTs alone. *Escherichia coli* was seen by both CMTs and BALF-wcDNA, and the remaining species detected by CMTs were those that both mNGS methods could detect (**Figure 1**). *Aspergillus fumigatus* was the most frequently reported fungus in all three methods.

34 patients had positive responsible pathogens according to CMTs (34/71, 47.89%), 58 and 51 patients were tested positive by BALF-cfDNA and BALF-wcDNA (BALF-cfDNA:58/71, 81.69%; BALF-wcDNA:51/71, 71.83%). The positive rate of BALF-mNGS was higher than CMTs (BALF-cfDNA vs. CMTs: 81.69% vs. 47.89%, P<0.001; BALF-wcDNA vs. CMTs: 71.83% vs. 47.89%, P=0.002; BALF-cfDNA vs. BALF-wcDNA: 81.69% vs. 71.83%, P=0.016).

31 patients were positive for the pathogens tested using both three methods (BALF-cfDNA, BALF-wcDNA, and CMTs), the consistency between the three methods was as follows: (1) matched in 7 (7/31, 22.58%) patients (perfect agreement in pathogens detection across all three methods), (2) partially matched in 17 (17/31, 54.84%) patients (at least one microorganism overlapped between three methods), (3) wholly mismatched in 7 (7/31, 22.58%) patients (no overlap of the pathogen between the three methods). 4 patients were not detected by 3 methods and 1 patient was detected only through CMTs.

### 3.3 Differences in numbers for RPM detected by BALF-cfDNA, BALF-wcDNA

**Figure 2:**
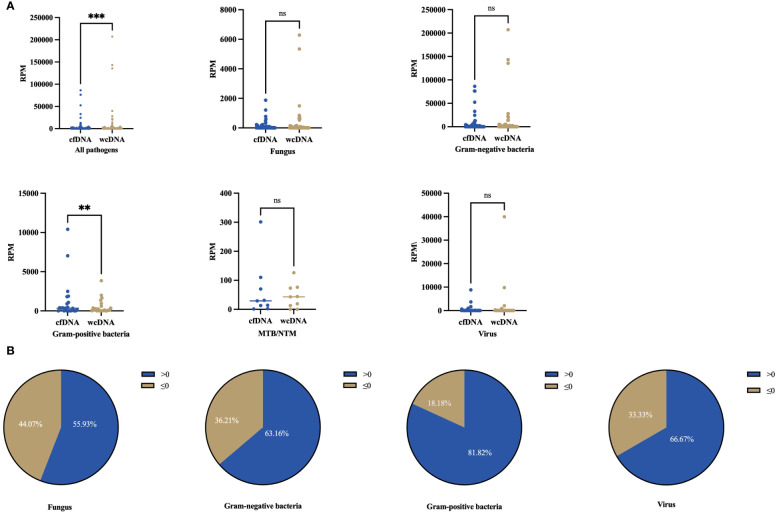
Differences in numbers for RPM detected by BALF-cfDNA, BALF-wcDNA. **(A)** Comparison of the number of RPM detected by BALF-cfDNA and BALF-wcDNA for all pathogens, Fungus, Gram-positive bacteria, Gram-negative bacteria, MTB/NTM, and virus. **(B)** >0 represented that the numbers of RPM detected by BALF-cfDNA were higher than that detected by BALF-wcDNA.≤0 represented that the numbers of RPM by BALF-wcDNA were higher or equal to BALF-cfDNA. **P<0.01; ***P<0.001; ns, no significant.

The RPM range observed was 1–86419 by BALF-cfDNA and 1–207274 by BALF-wcDNA. Generally, RPM detected by BALF-cfDNA was higher than those detected by BALF-wcDNA (29.5 vs. 19.5, P<0.001). For the Gram-positive bacteria, the RPM tested by BALF-cfDNA was greater than BALF-wcDNA (347.5 vs.119.5, P=0.008). Besides, for the detection of Gram-negative bacteria (188 vs. 97, P=0.071), fungus (17 vs. 9, P=0.467), MTB/NTM (29 vs.43, P=0.575), and virus (2 vs.2, P=0.163), there was no significant difference between BALF-cfDNA and BALF-wcDNA (**Figure 2**). These results reveal that mNGS of BALF-cfDNA captures more reads of microorganisms than mNGS of BALF-wcDNA.

### 3.4 Microbial distribution for pulmonary aspergillosis detected by BALF-cfDNA, BALF-wcDNA, and CMTs

**Figure 3:**
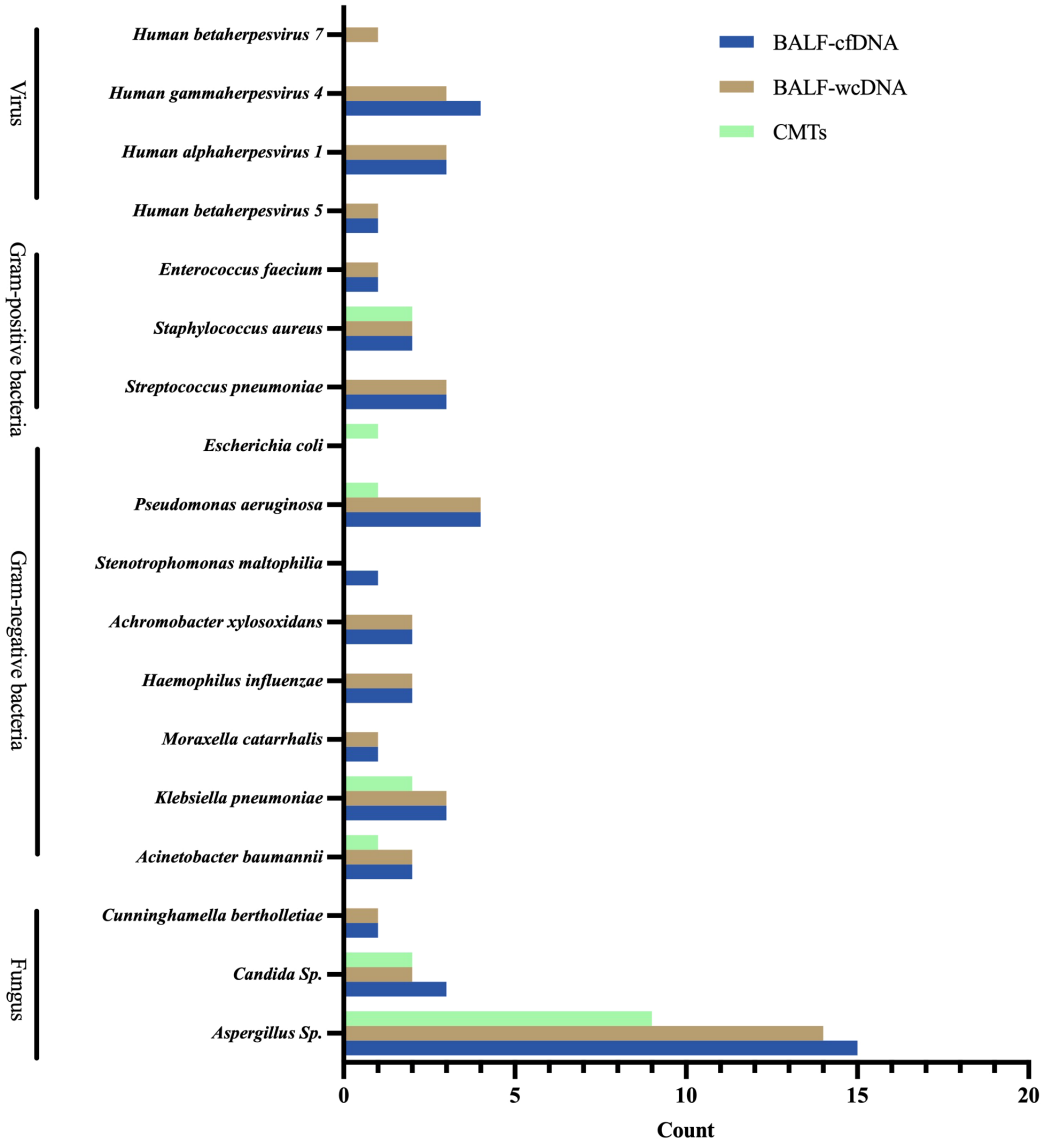
Microbial distribution for Pulmonary Aspergillus detected by BALF-cfDNA, BALF-wcDNA, and CMTs.

For PA patients, BALF-cfDNA identified 21 species (8 fungi, 10 bacteria, 3 viruses), BALF-wcDNA detected 21 species (8 fungi, 9 bacteria, 4 viruses), and CMTs detected 8 species (3 fungi, 5 bacteria) (**Figure 3**).

The positive rate for *Aspergillus* was 78.95% (15/19) for BALF-cfDNA and 73.68% (14/19) for BALF-wcDNA, there was no diversity in the positive rate (P=1.00). 9 patients were positive for *Aspergillus* by CMTs, with a positive rate of 47.37% (9/19), which was no statistical difference with BALF-cfDNA and BALF-wcDNA (P=0.109; 0.18). The number of *Aspergillus* identified by CMTs only and BALF-mNGS (cfDNA and wcDNA both) only were 2 and 8 patients, respectively, 1 patient detected *Aspergillus* by BALF-cfDNA only, 7 patients detected *Aspergillus* through three methods both.

*Aspergillus fumigatus*, closely followed by *Aspergillus flavus*, is the most common causative of *Aspergillus* in patients with PA. A higher number of RPM indicated *Aspergillus* detection through BALF-cfDNA instead of BALF-wcDNA (48 vs. 6, P=0.001).

### 3.5 Comparison of diagnostic performance among BALF-cfDNA, BALF-wcDNA, and CMTs in non-neutropenic pulmonary aspergillosis

Using a clinical diagnosis as the gold standard, we compared the diagnostic accuracy of detection methods in non-neutropenic PA (**Table 2**). BALF-cfDNA showed a sensitivity of 78.95% and a specificity of 84.62%, with PPV and NPV of 65.22% and 91.67%, respectively. The sensitivity, specificity, PPV, and NPV of BALF-wcDNA were 73.68%, 88.46%, 70.00%, and 90.20%. The sensitivity and specificity of BALF-cfDNA were similar to BALF-wcDNA. The sensitivity and specificity of CMTs in diagnosing PA were 47.37% and 84.62%, whereas the PPV and NPV were 52.94% and 81.48%.

**Table 2:** Diagnostic performance of BALF-cfDNA, BALF-wcDNA, and CMTs in PA.

Methods	Sensitivity (95%CI)	Specificity (95%CI)	PPV (95%CI)	NPV (95%CI)	AUC (95%CI)
BALF-cfDNA	78.95%	84.62%	65.22%	91.67%	0.818
	0.5667–0.9149	0.7248–0.9199	0.4489–0.8119	0.8045–0.9671	0.708–0.899
BALF-wcDNA	73.68%	88.46%	70.00%	90.20%	0.811
	0.5121–0.8819	0.7703–0.946	0.481–0.8545	0.79–2-0.9574	0.7–0.894
CMTs	47.37%	84.62%	52.94%	81.48%	0.66
	0.2733–0.6829	0.7284–0.9199	0.3096–0.7383	0.6916–0.8962	0.538–0.768
Culture	21.05%	96.15%	66.67%	76.92%	0.586
	0.0851–0.433	0.8702–0.9932	0.3000–0.9408	0.6536–0.8549	0.463–0.702
BALF-GM	31.58%	90.38%	54.55%	78.33%	0.61
	0.1536–0.5399	0.7939–0.9582	0.2801–0.7873	0.6638–0.8688	0.487–0.723
cfDNA+CMTs	89.47%	73.08%	54.84%	95.00%	0.813
	0.6861–0.9813	0.5975–0.8323	0.3777–0.7084	0.8350–0.9911	0.703–0.896
wcDNA+CMTs	84.21%	75.00%	55.17%	92.86%	0.796
	0.6243–0.9448	0.6179–0.8477	0.3755–0.7159	0.8099–0.9754	0.684–0.882

cfDNA, cell-free DNA metagenomic next generation sequencing; wcDNA, whole-cell DNA metagenomic next generation sequencing; CMTs, conventional microbiological tests; PPV, positive predictive value; NPV, negative predictive value; CI, confidence interval; AUC, the area under the ROC curve.

BALF-mNGS (cfDNA, wcDNA) perform better than culture or BALF-GM in sensitivity. No significant difference was observed in sensitivity and specificity between BALF-mNGS (cfDNA, wcDNA) and CMTs. Combination diagnosis of either positive for CMTs or BALF-mNGS (cfDNA, wcDNA) had significantly higher sensitivity, but significantly lower specificity than those of CMTs alone. (**Tables 2**, **3**).

**Table 3:** Comparison of the sensitivity, specificity, and ROC curve (AUC) among different diagnostic methods for suspected PA.

sensitivity/ specificity/AUC	BALF-cfDNA	BALF-wcDNA	CMTs	Culture
BALF-wcDNA	1.00/0.687/0.8412	/	/	/
CMTs	0.109/1.00/0.0602	0.18/0.774/0.0662	/	/
Culture	0.003/0.07/0.0018	0.006/0.219/0.0024	0.063/0.031/0.1911	/
BALF-GM	0.012/0.549/0.007	0.021/1.00/0.0088	0.25/0.25/0.2758	0.727/0.453/0.7651
cfDNA+CMTs	0.5/0.031/0.9053	/	0.008/0.031/0.0142	/
wcDNA+CMTs	/	0.5/0.016/0.7349	0.016/0.063/0.0244	/

**Figure 4:**
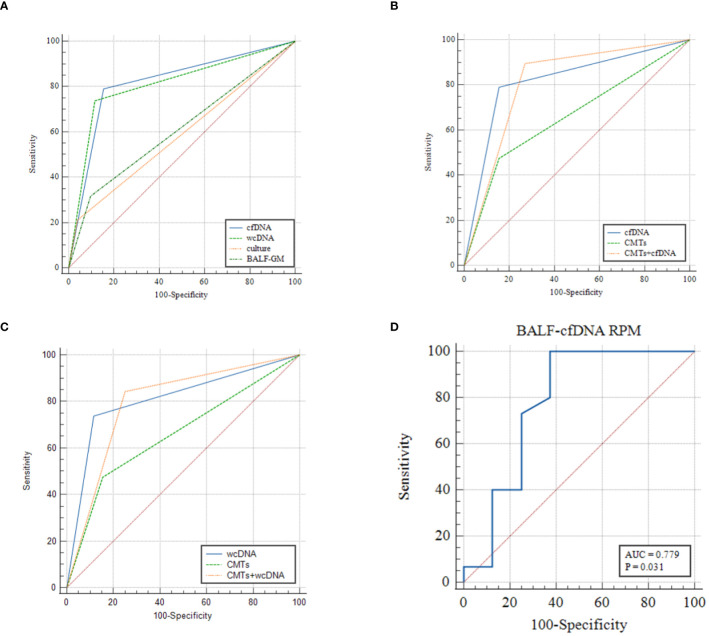
**(A–C)** ROC analysis of BALF-cfDNA, BALF-wcDNA, BALF-GM, culture and CMTs for the diagnosis of PA. **(D)** ROC analysis of RPM of Aspergillus detected by BALF-cfDNA in “True positive” PA.

ROC analysis of BALF-cfDNA, BALF-wcDNA, and CMTs for the diagnosis of PA yielded an AUC of 0.818, 0.811, and 0.66. BALF-mNGS (cfDNA, wcDNA) and CMTs exhibited comparable diagnostic abilities, while BALF-mNGS (cfDNA, wcDNA) outperformed culture or BALF-GM. The combination of BALF-mNGS (cfDNA, wcDNA) and CMTs is more effective than CMTs alone in the diagnosis of PA (**Figure 4**; **Table 3**).

14 out of 71 patients had taken antifungal agents (Voriconazole, Caspofungin) before providing a sample. For these patients, Sensitivity and specificity results indicate no significant differences when comparing BALF-mNGS (cfDNA, wcDNA) and CMTs for diagnosing PA (Sensitivity: BALF-cfDNA/BALF-wcDNA, 75.00% vs. 37.50%, P=0.25/75.00% vs. 37.50%, P=0.25; Specificity: BALF-cfDNA/BALF-wcDNA, 83.33% vs. 66.67%, P=1.00/100.00% vs. 66.67%). There is no notable difference in the diagnostic efficacy of BALF-mNGS (cfDNA, wcDNA) between this patient group and those who did not receive antifungal medication (BALF-cfDNA: sensitivity, 75.00% vs. 83.33%, P=1.00/specificity, 83.33% vs. 84.78%, P=1.00; BALF-wcDNA: sensitivity, 75.00% vs.75.00%, P=1.00/specificity,100.00% vs. 86.96%, P=1.00).

### 3.6 “True positive”, “False positive”, “False negative” by BALF-mNGS

**Figure 5:**
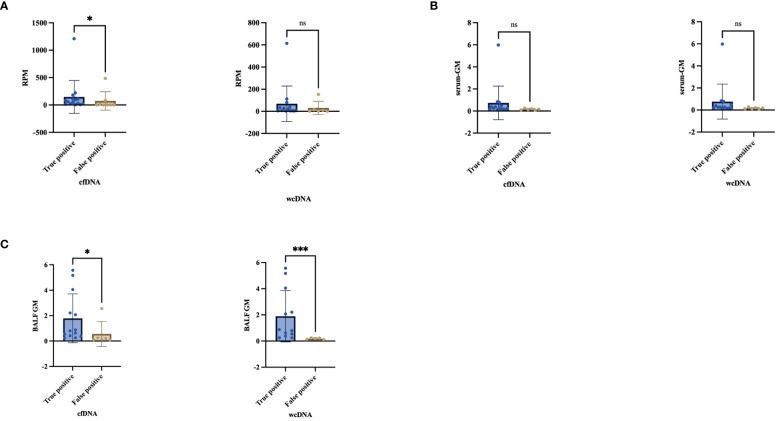
Comparison the difference between “True positive” and “False positive” by cfDNA, wcDNA. **(A)** The difference in the number of detected RPM between True positive and False positive. **(B, C)** Difference between serum-GM and BALF-GM in True positive and False positive. *P<0.05; ***P<0.001; ns, no significant.

We observed that the diversity of RPM for *Aspergillus* detected by BALF-cfDNA in “True positive” and “False positive” patients (61 vs. 2.5, P=0.03), whereas there was no significant difference in BALF-wcDNA (14 vs. 6, P=0.231). (**Figure 5**) We utilized the ROC curve to assess the diagnostic performance of BALF-cfDNA in “True positive” PA patients. The area under the ROC curve (AUC) for the RPM was 0.779 (P=0.031) (**Figure 4D**), and the cut-off value calculated according to the Yoden index was greater than 4.5, the sensitivity and specificity were 100.00% and 62.50%. For BALF-wcDNA, the AUC for the RPM was 0.673 (P=0.232).

The RPM of *Aspergillus* detected by BALF-cfDNA showed a positive correlation with BALF-GM (Spearman’s r values:0.481, P=0.037), while no correlation was observed with serum-GM. Next, we compared the levels of serum-GM and BALF-GM in patients categorized as “True positive” or “False positive” based on BALF-cfDNA and BALF-wcDNA separately, we found a difference in BALF-GM (BALF-cfDNA:0.8 vs.0.16, P=0.014; BALF-wcDNA:0.8 vs.0.17, P=0.002), but no significant difference in serum-GM (BALF-cfDNA:0.25 vs.0.15, P=0.057, BALF-wcDNA:0.25 vs. 0.11, P=0.053).

**Figure 6:**
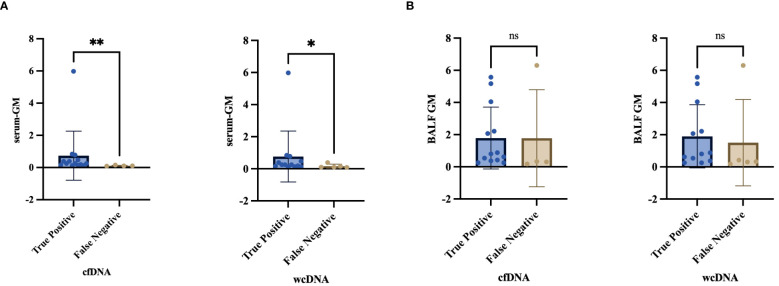
**(A, B)** Differential analysis of serum-GM, BALF-GM in the “True Positive” and “False Negative” PA by BALF-cfDNA, BALF-wcDNA. *P<0.05; **P<0.01; ns, no significant.

There was variety in serum-GM between PA patients with true-positive and false-negative by BALF-cfDNA (0.25 vs. 0.1, P=0.008) or BALF-wcDNA (0.25 vs. 0.1, P=0.034) separately, and no differences in BALF-GM (BALF-cfDNA:0.84 vs. 0.32, P=0.305; BALF-wcDNA:0.88 vs. 0.33, P=0.246) (**Figure 6**).

### 3.7 Antifungal agents’ application before enrollment

**Figure 7:**
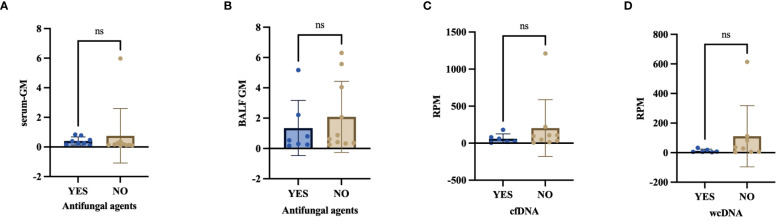
**(A–D)** Effect of antifungal agents on serum-GM, BALF-GM, RPM of Aspergillus detected by BALF-cfDNA and BALF-wcDNA. ns, no significant.

In our investigation, 8 out of 19 PA patients had received antifungal agents (Voriconazole, Caspofungin) before sample collection. 1 case (1/8, 12.50%) and 6 cases (6/8, 75.00%) were identified to have Aspergillus by CMTs and BALF-mNGS(cfDNA,wcDNA). We found no significant difference in the detection of *Aspergillus* among culture, CMTs, BALF-cfDNA, and BALF-wcDNA (P=0.344, 1.00, 0.065, 0.109) when treating PA patients with antifungal agents before sampling. In addition, there was also no diversity in serum GM (0.32 vs. 0.15, P=0.119), BALF-GM (0.54 vs. 0.75, P=0.354), RPM of *Aspergillus* detected by BALF-cfDNA (42 vs. 96, P=0.346) and BALF-wcDNA (6 vs. 32.5, P=0.154) (**Figure 7**).

### 3.8 Blood-cfDNA for suspected pulmonary aspergillosis

**Figure 8:**
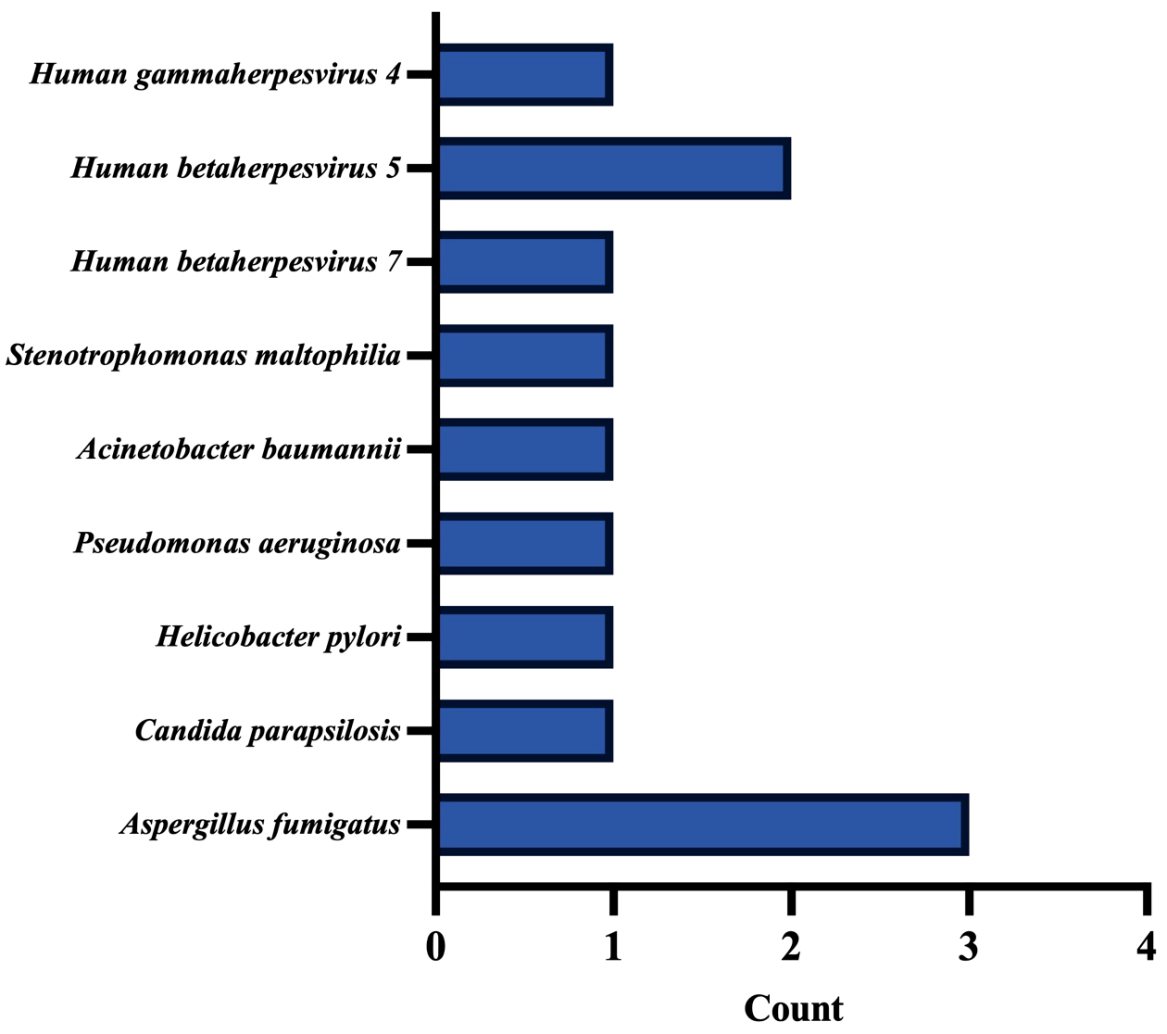
Pathogens distribution by blood cfDNA.

8 patients submitted both blood and BALF cfDNA, of whom 5 were diagnosed with PA (2 cases of CPA and 3 cases of IPA). However,4 of them did not detect any pathogens through blood-cfDNA. 3 patients (2 cases of IPA) detected *Aspergillus* through the blood cfDNA (**Figure 8**), and they also found Aspergills by BALF-mNGS (cfDNA, wcDNA).

A significant difference was observed in the serum-GM (0.78 vs.0.15, P=0.034) between the patients with positive and negative *Aspergillus* detection in blood-cfDNA, but there was no notable difference in BALF-GM (1.23 vs.0.62, P=1.00).

### 3.9 tNGS for suspected pulmonary aspergillosis

A total of 62 patients with suspected PA (18 PA and 44 non-PA) were subjected to tNGS. The pathogens detected are shown in **Supplementary Figure 1**.

The sensitivity and specificity of the diagnosis of PA by tNGS was 52.94% (0.3096–0.7383), 84.44% (0.7122–0.9225), and PPV and NPV were 56.25% (0.3318–0.769), and 82.61% (0.6928–0.9091), respectively.

The AUC values of tNGS, BALF-cfDNA, BALF-wcDNA and CMTs for PA in the 62 patients were re-analyzed using ROC curves, which yielded values of 0.716, 0.838, 0.808 and 0.658, respectively. The results demonstrated that tNGS did not exhibit a significant difference in comparison with the other methods (tNGS vs. BALF-cfDNA: 0.716 vs. 0.838, P=0.148; tNGS vs. BALF-wcDNA: 0.716 vs. 0.808, P=0.083; tNGS vs. CMTs: 0.716 vs. 0.658, P=0.521).

## 4 Discussion

This is the first report to assess the diagnostic efficacy of mNGS using cfDNA and wcDNA on BALF samples from patients with suspected PA. Our findings show a significantly higher detection rate of microorganisms in patients using BALF-mNGS (cfDNA, wcDNA) compared to CMTs. We provide evidence that BALF-mNGS might be the supplementary choice for the diagnosis of PA.

Rapid and precise identification of pathogens is hindered by the low sensitivity and time-consuming of conventional culture. Prior research indicates that mNGS permits impartial pathogen detection across diverse samples (Wei et al., 2022) and has outperformed culture in the investigation of infectious diseases (Zhang et al., 2020; Liang et al., 2022; Zuo et al., 2023). Our study demonstrates that BALF-mNGS (cfDNA, wcDNA) offers distinct advantages over culture in the diagnosis of PA with a high sensitivity. Conventional fungal and bacterial culture may overlook some pathogens due to differences in culture conditions, pathogen-specific incubation periods, and antimicrobial agents. Additionally, findings indicate that mNGS can promptly identify elusive pathogens that traditional culture methods may miss, possibly contributing to its superior sensitivity. The specificity of culture in diagnosing PA is not 100% and may be viewed as colonization or contamination. DNA extraction from certain PA patients was hindered by *Aspergillus*’ thick polysaccharide cell wall leading to negative mNGS results.

The diagnostic criteria for PA also include the GM test, which is frequently used clinically. GM test is an important mycologic evidence for diagnosing PA. Our findings indicate that BALF-mNGS has superior performance compared to BALF-GM for diagnosing PA. There have been limited studies comparing the diagnostic efficacy of BALF-mNGS with CMT in cases of PA. Thus, we conducted a comparison between the diagnostic performances of CMTs which includes culture and GM test to BALF-mNGS in diagnosing PA patients. While the sensitivity and specificity for CMTs were inferior to BALF-mNGS (cfDNA, wcDNA), there was no significant statistical difference between them. Combining a positive BALF-mNGS (cfDNA, wcDNA) or CMTs leads to a more effective diagnosis of PA than relying on CMTs alone, with higher sensitivity observed. This improvement can be attributed to the mNGS technique’s capacity to identify microorganisms that are challenging to cultivate. However, the specificity of this combination of diagnostics is significantly reduced for a number of reasons. These include contamination during sample processing and the detection of non-pathogenic microbial DNA by macrogenomic sequencing techniques. These factors may result in the generation of false-positive results, thereby reducing the specificity of the diagnosis. Considering the cost-effectiveness and clinical value, it is recommended that CMTs be the primary option for suspected PA. BALF-mNGS testing could serve as a complementary approach in cases where CMTs are negative despite strong suspicion of *Aspergillus* infection. In contrast, the value of BALF-mNGS combined with CMTs in patients with neutropenic PA remains to be evaluated. Previous studies have indicated that in immunocompromised IPA patients, the diagnostic performance of mNGS was significantly superior to that of CMTs (Shi et al., 2023). Furthermore, the diagnostic efficacy of mNGS in combination with CT was superior to that of immunocompetent patients (Zhan et al., 2023). It is therefore hypothesized that in patients with neutropenic, the performance of mNGS in combination with CMTs for the diagnosis of PA should remain superior to that of CMTs and perform better than in non-neutropenic patients. However, this needs to be confirmed by further studies.

cfDNA and wcDNA have different performances in pathogen recognition due to different nucleic acid extraction methods. mNGS of cfDNA uses host DNA degradation methods, which can lead to the loss of cfDNA from the supernatant and the potential introduction of reagent contamination (Ji et al., 2020). However, the extraction of wcDNA from BALF sample via cell fragmentation without host DNA degradation increases the release of human DNA. Previous study has reported that cfDNA outperformed wcDNA in patients with pulmonary or central nervous system infections (He et al., 2022; Yu et al., 2022). However, there was no statistical difference between BALF-cfDNA and BALF-wcDNA in diagnosing PA. Our study illustrates that BALF-cfDNA is superior to BALF-wcDNA in the detection rate of pathogens and assessing *Aspergillus* infection, with a higher RPM for the microorganism. The ratio of DNA in the sample determines the sensitivity of mNGS (Ebinger et al., 2021). mNGS of cfDNA directly extract DNA from the supernatant of BALF (Gu et al., 2021). Conversely, wcDNA does not filter human DNA from the supernatant, potentially resulting in a greater ratio of pathogenic DNA for cfDNA than wcDNA derived from the same BALF specimen. Besides, WcDNA may be better for the detection of the type of fungi. In this study, six microorganisms were exclusively detected by wcDNA, mostly fungi (5/6), and none had a high number of RPM detected. This occurrence may coincide with the fact that wcDNA extraction necessitates cell wall lysis and consequently detects more fungal species than cfDNA, or it may relate to sequencing contamination.

Plasma cell-free DNA sequencing has been widely used in clinical infectious diseases (Burnham et al., 2017; Armstrong et al., 2019). Consistent with the higher sensitivity of BALF-mNGS than blood-mNGS in patients with pneumonia as demonstrated by Chen et al. (Chen et al., 2020), our study similarly found that blood-mNGS was less sensitive than BALF-mNGS in detecting PA. Due to the limited number of blood samples in our study, we were unable to establish statistical significance in this comparison. We recruited the patients with CPA and IPA using blood-cfDNA. All patients with *Aspergillus* detected by blood-cfDNA were diagnosed with IPA except for one considered to be *Aspergillus* colonization. This could suggest that the blood-cfDNA is more suitable for patients with bloodstream rather than local infections, but further research is needed to confirm this. In contrast, the specificity of blood-mNGS in diagnosing PA was also lower than BALF-mNGS and CMTs in this study. This may be due to the small number of cases where blood-mNGS was used, and because *Aspergillus fumigatus* was detected in one patient by blood-mNGS who was later found to be colonized with *Aspergillus* following extensive clinical evaluation.

The most frequently detected pathogen was *Aspergillus fumigatus*, in agreement with previous research (Latgé and Chamilos, 2019). The identification of *Aspergillus* in respiratory samples does not necessarily indicate the presence of PA, as it is possible that the respiratory *Aspergillus* was colonized or contaminated during sequencing or other procedures. We try to distinguish the *Aspergillus* colonization by mNGS. Thus, patients with a confirmed PA diagnosis were identified as “True-positive” if *Aspergillus* was detected by the mNGS. Conversely, if the final diagnosis was non-PA but *Aspergillus* was detected by the mNGS, patients were classified as “False-positive”. Additionally, a patient was labeled as “False-negative” if *Aspergillus* was not detected by the mNGS while they had a PA diagnosis.

The GM test identifies a polysaccharide antigen present in the cell wall of Aspergillus. The antigen can be released early during *Aspergillus* tissue invasion from the outer layer of the cell wall into the bloodstream, and can thus be detected in bodily fluids. The degree of fungal growth is reflected by the amount of antigen released, which in turn indicates the severity of the infection. In the investigation of patients with central nervous system infections, pathogen reads from mNGS cohered with modifications in cerebrospinal fluid WBC, exhibiting a connection between the number of pathogen readings and the extent of the disease’s infection (Zhang et al., 2020). In this study, BALF-GM levels showed a positive correlation with the RPM of *Aspergillus*, true-positive patients had higher serum-GM levels than false-negative patients. Furthermore, serum-GM levels were found to be higher in individuals with positive blood-cfDNA for *Aspergillus* as compared to those with negative blood-cfDNA. This indirectly suggests that the RPM of Aspergillus in this study reflects the fungal loads. In several studies (Zhou et al., 2017; Sehgal et al., 2019; Dai et al., 2021), BALF-GM proved more effective than serum-GM in diagnosing non-neutropenic PA. Our study found BALF-GM to be more relevant than serum-GM in differentiating true-positive from false-positive patients. Wang et al (Wang et al., 2022), also discovered that true-positive patients with microorganisms detected by mNGS had more reads than false-positive patients in invasive fungal disease. Our previous study (Liu et al., 2021) demonstrates the capacity to distinguish Pneumocystis jirovecii Pneumonia and Pneumocystis jirovecii Colonization through pathogen reads using mNGS. In our study, true-positive patients displayed a greater RPM of *Aspergillus* than false-positive patients when utilizing BALF-cfDNA and the cut-off value was 4.5, whereas BALF-wcDNA was not significantly different. These findings suggest that BALF-cfDNA may be a more appropriate test for patients suspected to have PA than BALF-wcDNA. It appears that *Aspergillus* infection status can be inferred from the high or low RPM count.

Previous studies (Chen et al., 2020) have highlighted that using antimicrobial drugs prior to specimen collection can lead to inaccurate results in false-negative results in microbial cultures. Besides, Qing Miao et al. (Miao et al., 2018) demonstrated that mNGS is less susceptible to the effects of prior antibiotic exposure. Our study discovered a negligible impact of antifungal drug exposure on culture outcomes, CMTs, and the detection of *Aspergillus* by BALF-mNGS (cfDNA, wcDNA). This may be the reason for the shorter duration of antifungal drug use. It could also be due to the lower sensitivity of fungal cultures compared to bacterial cultures, resulting in antifungal drugs having a weaker effect. However, larger samples are needed to confirm this.

In this study, we also employed tNGS to assess its efficacy in diagnosing PA. tNGS represents a novel approach that combines the advantages of PCR and NGS. This technology amplifies target pathogen sequences by PCR, thereby reducing host nucleic acid interference and enhancing detection sensitivity. The results demonstrated that tNGS exhibited lower sensitivity values than BALF-mNGS in the diagnosis of PA. However, tNGS demonstrated comparable sensitivity to CMTs. This may be attributed to the fact that the tNGS technique employed in this study is limited to the detection of *Aspergillus fumigatus* and is unable to identify other types of *Aspergillus*, including *Aspergillus flavus* and *Aspergillus niger*, among others. The ROC curves also demonstrated superior diagnostic performance of the BALF-mNGS compared to tNGS, although the difference was not statistically significant. To date, no studies have been conducted to assess the applicability of tNGS in the context of non-neutropenic PA. Consequently, further studies with larger sample sizes are required to investigate the potential of tNGS in this area.

Our study has several limitations. Firstly, the sample size in this study was limited. In future studies, the sample size will be expanded in order to further stratify IPA and CPA, thereby providing more valuable information for the diagnosis and treatment of both diseases. Secondly, we were unable to retain blood from all enrolled patients at the same time as the BALF samples for simultaneous mNGS test, which prevented us from better assessing its diagnostic efficacy. Thirdly, due to COVID-19, the BALF sample could not be transported to the business lab for mNGS testing in time, which prevented the results from serving as a recommendation for using antimicrobial drugs.

## 5 Conclusion

In summary, the results indicate that BALF-mNGS (cfDNA, wcDNA) performs better than CMTs in detecting pathogens. Furthermore, when diagnosing non-neutropenic PA, BALF-mNGS (cfDNA, wcDNA) shows similarity to CMTs but superiority to culture and BALF-GM. Combining BALF-mNGS (cfDNA, wcDNA) with CMTs shows the potential to enhance the sensitivity of diagnostic performance. Additionally, the RPM of *Aspergillus* serves as an indicator of fungal loads. An additional advantage of BALF-cfDNA over BALF-wcDNA is the ability to differentiate between “True positive” and “False positive” patients with PA. Therefore, mNGS of BALF-cfDNA may present a novel diagnostic technology for PA.

## Data availability statement

The data presented in the study are deposited in the National Genomics Data Center (https://ngdc.cncb.ac.cn/?lang=zh) repository, accession number PRJCA028215.

## Ethics statement

The studies involving humans were approved by Ethics Committee of the Nanjing Jingling Hospital. The studies were conducted in accordance with the local legislation and institutional requirements. The participants provided their written informed consent to participate in this study.

## Author contributions

XC: Data curation, Formal analysis, Writing – original draft. CS: Data curation, Writing – review & editing, Formal analysis. HZ: Data curation, Writing – review & editing. YC: Data curation, Writing – review & editing. MC: Data curation, Writing – review & editing. LW: Data curation, Validation, Writing – review & editing. WS: Data curation, Writing – review & editing. YT: Data curation, Writing – review & editing. GM: Data curation, Writing – review & editing. BH: Data curation, Writing – review & editing. SY: Data curation, Writing – review & editing. JZ: Data curation, Writing – review & editing. JW: Data curation, Writing – review & editing. YJL: Data curation, Writing – review & editing. YG: Methodology, Writing – review & editing. MS: Data curation, Writing – review & editing. YW: Data curation, Writing – review & editing. YYL: Data curation, Writing – review & editing. XS: Funding acquisition, Writing – review & editing.
