# Supplementary material for: The value of metagenomic next-generation sequencing with different nucleic acid extracting methods of cell-free DNA or whole-cell DNA in the diagnosis of non-neutropenic pulmonary aspergillosis
Source: Front Cell Infect Microbiol. 2024 Jul 29;14:1398190. doi: 10.3389/fcimb.2024.1398190 (PMC11317373; doi:10.3389/fcimb.2024.1398190)
Supplement: Supplementary file 1 [file DataSheet_1.docx]

Supplementary Material

The value of metagenomic next-generation sequencing with different nucleic acid extracting methods of cell-free DNA or whole-cell DNA in the diagnosis of non-neutropenic pulmonary aspergillosis

**Xiaomin Cai^1,2, †^, Chao Sun^2,^** **^†^, Huanhuan Zhong^2,8^, Yuchen Cai^2^, Min Cao^1^, Li Wang^4^, Wenkui Sun^5^, Yujian Tao^6^, Guoer Ma^7^, Baoju Huang^4^, Shengmei Yan^2^, Jinjin Zhong^2^, Jiamei Wang^3^, Yajie Lu^2^, Yuanlin Guan^9^, Mengyue Song^3^, Yujie Wang^2^, Yuanyuan Li^2^, Xin Su^1,2*^**

^1^Department of Respiratory and Critical Care Medicine, Nanjing Drum Tower Hospital, Affiliated Hospital of Medical School, Nanjing University, Nanjing, China

^2^Department of Respiratory and Critical Medicine, Jinling Hospital, Affiliated Hospital of Medical School, Nanjing University, Nanjing, China.

^3^Department of Respiratory and Critical Care Medicine, Jinling Hospital, Nanjing Medical University, Nanjing, China

^4^Department of Respiratory and Critical Care Medicine, The Second Affiliated Hospital of Nanjing University of Chinese Medicine, Nanjing, China

^5^Department of Respiratory and Critical Care Medicine, Jiangsu Province Hospital, The First Affiliated of Nanjing Medical University, Nanjing, China

^6^Department of Respiratory and Critical Care Medicine, Affiliated Hospital of Yangzhou University, Yangzhou, China

^7^Department of Respiratory and Critical Care Medicine, Affiliated Hospital of Jiangsu University, Zhenjiang, China

^8^Department of Respiratory and Critical Care Medicine, The Second Affiliated Hospital of Suzhou University, Suzhou, China

^9^Department of Research and Development, Hugobiotech Co., Ltd., Beijing, China

†These authors contributed equally to this work and share first authorship

*** Correspondence:** Corresponding Author: [suxinjs@163.com](mailto:suxinjs@163.com)

# Supplementary Table 1 Pathogens detectable by tNGS

| Gram-positive bacteria | Gram-negative bacteria | Mycoplasma/Chlamydi | Virus | Fungus | Rickettsia |
| --- | --- | --- | --- | --- | --- |
| Streptococcus pneumoniae | Neisseria meningitidis | mycoplasma pneumoniae | Human bocaparvovirus 1 | Cryptococcus neoformans | Rickettsia typhi |
| Mycolicibacterium tuberculosis complex | Klebsiella oxytoca | Chlamydia pneumoniae | Human bocaparvovirus 4 | Candida albicans | Orientia tsutsugamushi |
| Corynebacterium diphtheriae | Stenotrophomonas maltophilia | Ureaplasma parvum | Human herpesvirus 2 | Talaromyces mameffei | Rickettsia prowazekii |
| Nocardia brasiliensis | Acinetobacter baumannii | Chlamydia trachomatis | Human herpesvirus 5 | Rhizopus microsporus | Coxiella burnetii |
| Arcanobacterium pyogenes | Escherichia coli | Ureaplasma urealyticum | Polyomavirus BK | Rhizopus oryzae | Rickettsia rickettsiae |
| Bacillus cereus | Bacteroides fragilis | Chlamydia psittaci | Human bocaparvovirus 2 | Aspergillus fumigatus | |
| Nontuberculosis mycobacteria | Bordetella parapertussis | | Human parvovirus B19 | Cyptococcus gattii | |
| Streptococcus pyogene | Pasteurella multocida | | Human Alphaherpesvirus 3 | Rhizomucor pusillus | |
| Staphylococcus aureus | Burkholderia anthracis | | Human herpes virus 6 | Scedosperium apiospermum | |
| Mycolicibacterium tuberculosis complex | Serratia marcescens | | JC polyomavirus | Trichosporon asahii | |
| Nocardiosis | Moraxella catarrhalis | | Human bocaparvovirus 3 | Pneumocystis jiroveci | |
| Corynebacterium pseudotuberculosis | Bordetella pertussis | | Human alphaherpesvirus 1 | Histoplasma capsulatum | |
| Tropheryma whipplei | Pseudomonas aeruginosa | | Human herpesvirus 4 | Lichtheimia corymbifera | |
| Enterococcus faecium | Klebsiella aerogenes | | Human betaherpesvirus 7 | Schizophylla |  |
| Streptococcus agalactiae | Burkholderia cepacia | | Human adenovirus | |  |
| Star-shaped Nocardia | Bordetella holmesii | | Influenza B virus | |  |
| Nocardia cyriacigeorgica | Francisella tularensis | | Human coronavirus 229E | |  |
| Bacillus anthracis | Burkholderia pseudomallei | | Human coronavirus OC43 | |  |
| Rhodococcus equi | Haemophilus influenzae | | Human parainfluenza virus-3 | |  |
|  | Legionella pneumophila | | Respiratory syncytial virus B | |  |
|  | Klebsiella pneumoniae | | Measles virus |  |  |
|  | Haemophilus haemolyticus | | Enterovirus |  |  |
|  | Enterobacter cloacae complex | | Influenza C virus | |  |
|  | Bba -Bartonella bacilliformis | | Human coronavirus HKU1 | |  |
|  | Salmonella enterica | | Human parainfluenza virus 1 | |  |
|  | Proteus mirabilis | | Human parainfluenza virus 4 | |  |
|  | Elizabethkingia meningosepticum | | Mumps virus |  |  |
|  |  |  | Rotavirus |  |  |
|  |  |  | Rhinovirus |  |  |
|  |  |  | Human metapneumovirus | |  |
|  |  |  | Human coronavirus NL63 | |  |
|  |  |  | Human parainfluenza Virus 2 | |  |
|  |  |  | Respiratory syncytial virus A | |  |
|  |  |  | Rubella virus |  |  |
|  |  |  | Influenza a virus | |  |


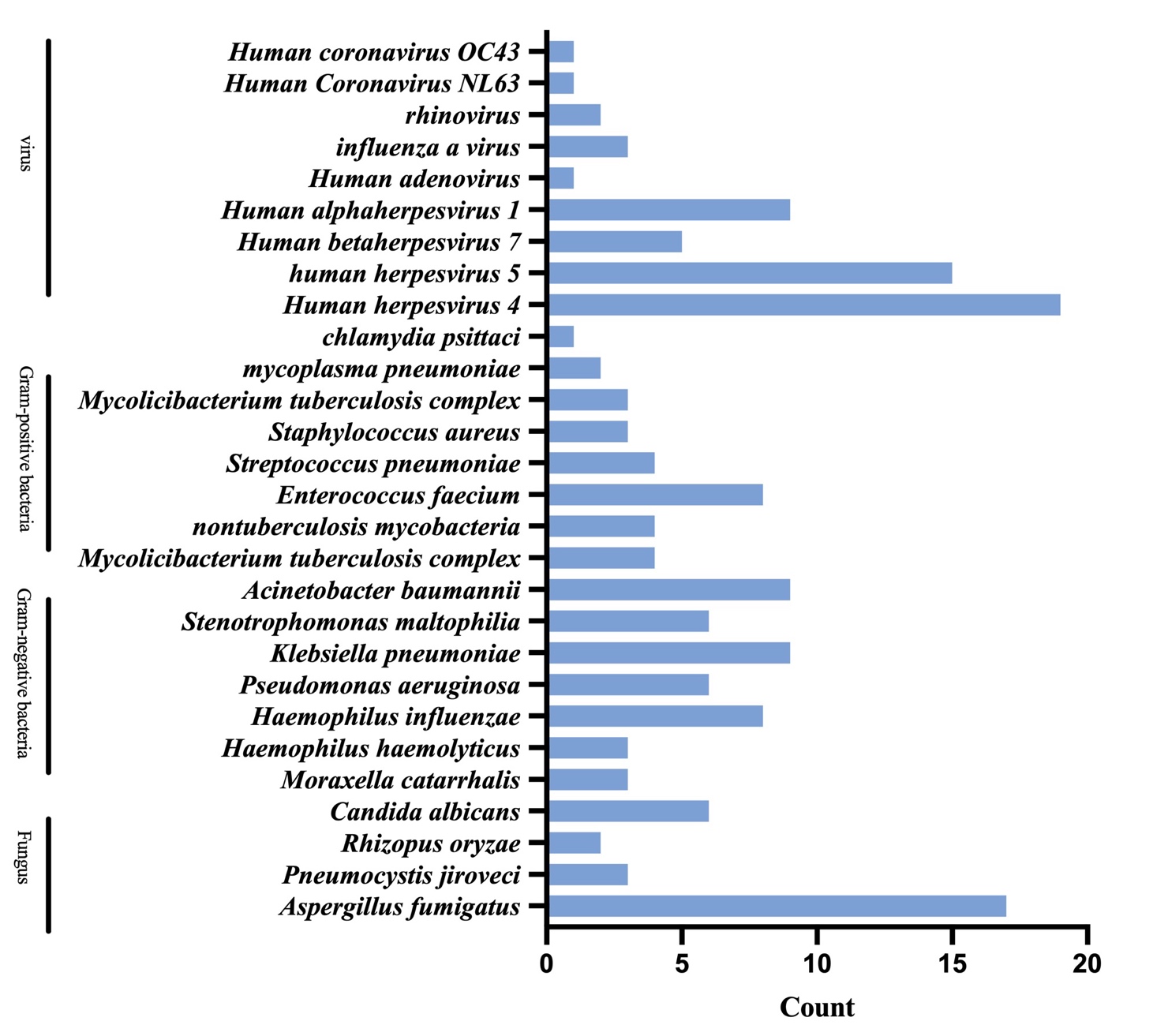


**Supplementary Figure 1.** Microbial distribution for suspected Pulmonary Aspergillus detected by tNGS.
